# Methodological challenges and solutions in auditory functional magnetic resonance imaging

**DOI:** 10.3389/fnins.2014.00253

**Published:** 2014-08-21

**Authors:** Jonathan E. Peelle

**Affiliations:** Department of Otolaryngology, Washington University in St. LouisSt. Louis, MO, USA

**Keywords:** auditory cortex, auditory perception, speech, music, hearing, executive function

## Abstract

Functional magnetic resonance imaging (fMRI) studies involve substantial acoustic noise. This review covers the difficulties posed by such noise for auditory neuroscience, as well as a number of possible solutions that have emerged. Acoustic noise can affect the processing of auditory stimuli by making them inaudible or unintelligible, and can result in reduced sensitivity to auditory activation in auditory cortex. Equally importantly, acoustic noise may also lead to increased listening effort, meaning that even when auditory stimuli are perceived, neural processing may differ from when the same stimuli are presented in quiet. These and other challenges have motivated a number of approaches for collecting auditory fMRI data. Although using a continuous echoplanar imaging (EPI) sequence provides high quality imaging data, these data may also be contaminated by background acoustic noise. Traditional sparse imaging has the advantage of avoiding acoustic noise during stimulus presentation, but at a cost of reduced temporal resolution. Recently, three classes of techniques have been developed to circumvent these limitations. The first is Interleaved Silent Steady State (ISSS) imaging, a variation of sparse imaging that involves collecting multiple volumes following a silent period while maintaining steady-state longitudinal magnetization. The second involves active noise control to limit the impact of acoustic scanner noise. Finally, novel MRI sequences that reduce the amount of acoustic noise produced during fMRI make the use of continuous scanning a more practical option. Together these advances provide unprecedented opportunities for researchers to collect high-quality data of hemodynamic responses to auditory stimuli using fMRI.

## Introduction

Over the past 20 years, functional magnetic resonance imaging (fMRI) has become the workhorse of cognitive scientists interested in noninvasively measuring localized human brain activity. Although the benefits provided by fMRI have been substantial, there are numerous ways in which it remains an imperfect technique. This is perhaps nowhere more true than in the field of auditory neuroscience due to the substantial acoustic noise generated by standard fMRI sequences. In order to study brain function using fMRI, auditory researchers face what can seem like an unappealing array of methodological decisions that impact the acoustic soundscape, cognitive performance, and imaging data characteristics to varying degrees. Here I review the challenges faced in auditory fMRI studies, possible solutions, and prospects for future improvement. Much of the information regarding the basic mechanics of noise in fMRI can be found in previous reviews (Amaro et al., 2002; Moelker and Pattynama, 2003; Talavage et al., 2014); although I have repeated the main points for completeness, I focus on more recent theoretical perspectives and methodological advances.

## Sources of acoustic interference in fMRI

Table 1 summarizes several factors that contribute to the degradation of acoustic signals during fMRI. Echoplanar imaging (EPI) sequences commonly used to detect the blood oxygen level dependent (BOLD) signal in fMRI require radiofrequency (RF) pulses that excite tissue and gradient coils that help encode spatial position by altering the local magnetic field. During EPI the gradient coils switch between phase encoding and readout currents, producing Lorentz forces that act on the coils and connecting wires. These vibrations travel as compressional waves through the scanner hardware and eventually enter the air as acoustic sound. This gradient-induced vibration produces the most prominent acoustic noise during fMRI, and can continue for up to approximately 0.5 s after the gradient activity ceases (Ravicz et al., 2000). Because the Lorentz force is proportional to the main magnetic field strength (*B*_0_) and the gradient current, both high *B*_0_ and high gradient amplitudes generally increase the amount of acoustic noise generated (Moelker et al., 2003). For example, increasing field strength from 0.2 to 3 T will bring maximum acoustic noise from ~85 to ~130 dB SPL (Foster et al., 2000; Ravicz et al., 2000; Price et al., 2001).

**Table 1:** **Sources of acoustic interference during fMRI**.

**Source**	**Approximate noise level (dB SPL)**
Gradient coils	85–130
Helium pump and air circulating	57–76
In-ear foam earplugs	–
Sub-optimal headphones	–

Although the noise generated by gradient switching is the most obvious (i.e., loudest) source of acoustic noise during fMRI, it is not the only source of acoustic interference. RF pulses contribute additional acoustic noise, and noise is also present as a result of air circulation systems and helium pumps in the range of 57–76 dB SPL (Ravicz et al., 2000). Because RF and helium pump noise is substantially quieter than that generated by gradient coils it probably provides a negligible contribution when scanning is continuous, but may be more relevant in sparse or interleaved silent steady state (ISSS) imaging sequences (described in a later section) when gradient-switching noise is absent. Auditory clarity can also be reduced as a result of in-ear hearing protection and sub-optimal headphone systems.

Separately or together, these noise sources provide a level of acoustic interference that is significantly higher than that found in a typical behavioral testing environment. In the next section I turn to the more interesting question of the various ways in which this cacophony may impact auditory neuroscience.

## Challenges of acoustic noise in auditory fMRI

**Figure 1:**
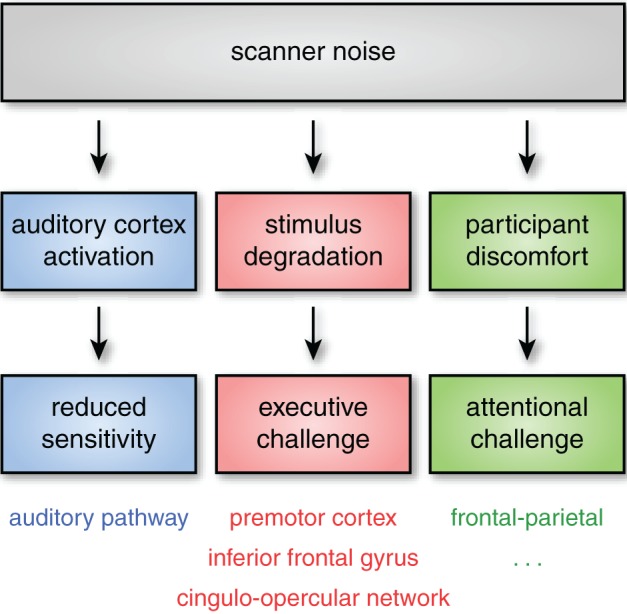
**Even when subjects can hear stimuli, acoustic noise can impact neural activity through at least three pathways**. First, acoustic noise from the scanner stimulates the auditory pathway (including auditory cortex), reducing sensitivity to experimental stimuli. Second, successfully processing degraded stimuli may require additional executive processes (such as verbal working memory or performance monitoring). These executive processes are frequently found to rely on regions of frontal and premotor cortex, as well as the cingulo-opercular network. Finally, scanner noise may increase attentional demands, even for non-auditory tasks, an effect that is likely exacerbated in more sensitive subject populations. Although the specific cognitive and neural consequences of these challenges may vary, the critical point is that scanner noise can alter both cognitive demand and the patterns of brain activity observed through multiple mechanisms, affecting both auditory and non-auditory brain networks.

Acoustic noise can influence neural response through at least three independent pathways, illustrated schematically in Figure 1. The effects will vary depending on the specific stimuli, population being studied, and brain networks being examined. Importantly, though, in many cases the impact of noise on brain activation can be seen outside of auditory cortex. In this section I review the most pertinent challenges caused by acoustic scanner noise.

### Energetic masking

Energetic masking refers to the masking of a target sound by a noise or distractor sound that obscures information in the target. That is, interference occurs at a peripheral level of processing, with the masker already obscuring the target as the sound enters the eardrum (and thus at the most peripheral levels of the auditory system). The level of masking is often characterized by the signal-to-noise ratio (SNR), which reflects the relative loudness of the signal and masker. For example, an SNR of +5 dB indicates that on average the target signal is 5 dB louder than the masker. If scanner noise at a subject's ear is 80 dB SPL, achieving a moderately clear SNR of +5 would require presenting a target signal at 85 dB SPL. When considering the masking effects of noise it is important to note that the characteristics of the noise are also important: noise that has temporal modulation can permit listeners to glean information from the “dips” in the noise masker.

Energetic masking highlights the most obvious challenge of using auditory stimuli in fMRI: Subjects may not be able to perceive auditory stimuli due to scanner noise. If stimuli are inaudible—or less than fully perceived in some way—interpreting the subsequent neural responses can be problematic. A different (but related) sort of energetic masking challenge arises in experiments in which subjects are required to make vocal responses, as scanner noise can interfere with an experimenter's understanding of subject responses; in some cases this can be ameliorated by offline noise reduction approaches (e.g., Cusack et al., 2005). In addition, the presence of acoustic noise may also change the quality of vocalizations produced by subjects (Junqua, 1996). Acoustic noise thus impacts not only auditory perception, but speech production, which may be important for some experimental paradigms.

Two ways of ascertaining the degree to which energetic masking is a problem are (1) to ask participants about their subjective experience hearing stimuli or (2) to include a discrimination or recall test that can empirically verify the degree to which auditory stimuli are perceived. Given individual differences in hearing level and ability to comprehend stimuli in noise, these are likely best done for each subject, rather than, for example, audibility being verified solely by the experimenter. It is also important to test audibility using stimuli representative of those used in the experiment, as the masking effects of scanner noise can be influenced by specific acoustic characteristics of the target stimuli (for example, being more detrimental to perception of birdsong than speech).

Although it is naturally important for subjects to be able to hear experimental stimuli (and for experimenters to hear subject responses, if necessary), the requirement of audibility is obvious enough that it is often taken into account when designing a study. However, acoustic noise may also cause more pernicious challenges, to which I turn in the following sections.

### Auditory activation

A natural concern regarding acoustic noise during fMRI relates to the activation along the auditory pathway resulting from the scanner noise. If brain activity is modulated in response to scanner noise, might this reduce our ability to detect signals of interest? To investigate the effect of scanner noise on auditory activation, Bandettini et al. (1998) acquired data with and without EPI-based acoustic stimulation, enabling them to compare brain activity that could be attributed to scanner noise. They found that scanner noise results in increased activity bilaterally in superior temporal cortex (see also Talavage et al., 1999). Notably, this activity was not observed only in primary auditory cortex, but in secondary auditory regions as well. The timecourse of activation to scanner noise peaks 4–5 s after stimulus onset, returning to baseline by 9–12 s (Hall et al., 2000), and is thus comparable to that observed in other regions of cortex (Aguirre et al., 1998). Scanner-related activation in primary and secondary auditory cortex limits the dynamic range of these regions, producing weaker responses to auditory stimuli (Shah et al., 1999; Talavage and Edmister, 2004; Langers et al., 2005; Gaab et al., 2007). In addition to overall changes in magnitude or spatial extent of auditory activation, scanner noise can affect the level at which stimuli need to be presented for audibility, which can in turn affect activity down to the level of tonotopic organization (Langers and van Dijk, 2012). Thus, if activity along the auditory pathway proper is of interest, the contribution of scanner noise must be carefully considered when interpreting results.

It is worth noting that while previous studies have investigated the effect of scanner noise on overall (univariate) response magnitude, the degree to which this overall change in gain may affect multivariate analyses is unclear. Again, this is true for activity in both auditory cortex and regions further along the auditory processing hierarchy (Davis and Johnsrude, 2007; Peelle et al., 2010b).

### Cognitive effort during auditory processing

Although acoustic noise can potentially affect all auditory processing, most of the research on the cognitive effects of acoustic challenge has occurred in the context of speech comprehension. There is increasing consensus that decreased acoustic clarity requires listeners to engage additional cognitive processing to successfully understand spoken language. For example, after hearing a list of spoken words, memory is worse for words presented in noise, even though the words themselves are intelligible (Rabbitt, 1968). When some words are presented in noise (but are still intelligible), subjects have difficulty remembering not only the words in noise, but prior words (Rabbitt, 1968; Cousins et al., 2014), suggesting an increase in cognitive processing for degraded speech that lasts longer than the degraded stimulus itself and interferes with memory (Miller and Wingfield, 2010). Additional evidence supporting the link between acoustic challenge and cognitive resources comes from pupillometry (Kuchinsky et al., 2013; Zekveld and Kramer, 2014) and visual tasks which relate to individual differences in speech perception ability (Zekveld et al., 2007; Besser et al., 2012). The additional cognitive resources required are not specific to acoustic processing but appear to reflect more domain-general processes (such as verbal working memory) recruited to help with auditory processing (Wingfield et al., 2005; Rönnberg et al., 2013). Thus, acoustic challenge can indirectly impact a wide range of cognitive operations.

Consistent with this shared resource view, behavioral effects of acoustic clarity are reliably found on a variety of tasks. Van Engen et al. (2012) compared listeners' recognition memory for sentences spoken in conversational speech compared to those spoken in a clear speaking style (with accentuated acoustic features), and found that memory was superior for the acoustically-clearer sentences. Likewise, listeners facing acoustic challenge—due to background noise, degraded speech, or hearing impairment—perform poorer than listeners with normal hearing on auditory tasks ranging from sentence processing to episodic memory tasks (Pichora-Fuller et al., 1995; Surprenant, 1999; Murphy et al., 2000; McCoy et al., 2005; Tun et al., 2010; Heinrich and Schneider, 2011; Lash et al., 2013).

**Figure 2:**
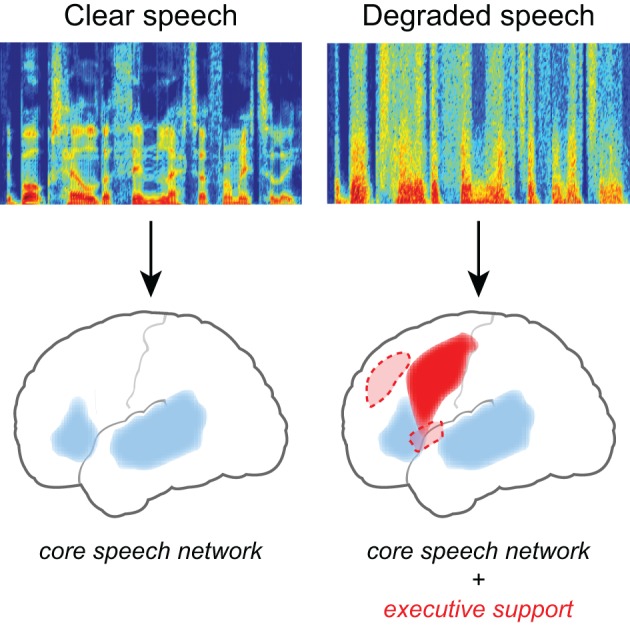
**Listening to degraded speech requires increased reliance on executive processing and a more extensive network of brain regions**. When speech clarity is high, neural activity is largely confined to traditional frontotemporal “language” regions including bilateral temporal cortex and left inferior frontal gyrus. When speech clarity is reduced, additional activity is frequently seen in frontal cortex, including middle frontal gyrus, premotor cortex, and the cingulo-opercular network (consisting of bilateral frontal operculum and anterior insula, as well as dorsal anterior cingulate) (Dosenbach et al., 2008).

Converging evidence for the neural effects of effortful listening comes from fMRI studies in which increased neural activity is seen for degraded speech relative to unprocessed speech (Scott and McGettigan, 2013), illustrated in Figure 2. Davis and Johnsrude (2003) presented listeners with sentences that varied in their intelligibility, with speech clarity ranging from unintelligible to fully intelligible. They found greater activity for degraded speech compared to fully intelligible speech in the left hemisphere, along both left superior temporal gyrus and inferior frontal cortex. Importantly, increased activity in frontal and prefrontal cortex was greater for moderately distorted speech than either fully intelligible or fully unintelligible speech (i.e., an inverted U-shaped function), consistent with its involvement in recovering meaning from degraded speech (as distinct from a simple acoustic response). Acoustic clarity (i.e., SNR) also impacts the brain networks supporting semantic processing during sentence comprehension (Davis et al., 2011), possibly reflecting increased use of semantic context as top-down knowledge during degraded speech processing (Obleser et al., 2007; Obleser and Kotz, 2010; Sohoglu et al., 2012).

Additional studies using various forms of degraded speech have also found difficulty-related increases in regions often associated with cognitive control or performance monitoring, such as bilateral insula and anterior cingulate cortex (Eckert et al., 2009; Adank, 2012; Wild et al., 2012; Erb et al., 2013; Vaden et al., 2013). The stimuli used in these studies are typically less intelligible than unprocessed speech (e.g., 4- or 6-channel vocoded^1^ speech, or low-pass filtered speech). Thus, although the increased recruitment of cognitive and neural resources to handle degraded speech is frequently observed, the specific cognitive processes engaged—and thus the pattern of neural activity—depend on the degree of acoustic challenge presented. An implication of this variability is that it may be hard to predict *a priori* the effect of acoustic challenge on the particular cognitive system(s) of interest.

In summary, there is clear evidence that listening to degraded speech results in increased cognitive demand and altered patterns of brain activity. The specific differences in neural activity depend on the degree of the acoustic challenge, and thus may differ between moderate levels of degradation (when comprehension accuracy remains high and few errors are made) and more severe levels of degradation (when comprehension is significantly decreased). It is important to note that effort-related differences in brain activity can be seen both within the classic speech comprehension network and in regions less typically associated with speech comprehension, and depend on the nature of both the stimuli and the task. Furthermore, the way in which these effort-related increases interact with other task manipulations has received little empirical attention, and thus the degree to which background noise may influence observed patterns of neural response for many specific tasks is largely unknown.

Finally, although most of the research on listening effort has been focused on speech comprehension, it is reasonable to think that many of these same principles might transfer to other auditory domains, such as music or environmental sounds. And, as covered in the next section, effects of acoustic challenge need not even be limited to auditory tasks.

### Effects of acoustic noise in non-auditory tasks

Although the interference caused by acoustic noise is most obvious when considering auditory tasks, it may also affect subjects' performance on non-auditory tasks (for example, by increasing demands on attention systems). The degree to which noise impacts non-auditory tasks is an important one for cognitive neuroscience. Unfortunately, there have been relatively few studies addressing this topic directly.

Using continuous EPI, Cho et al. (1998a) had subjects perform simple tasks in the visual (flickering checkerboard) and motor (finger tapping) domains, with and without additional scanner noise played through headphones. The authors found opposite effects in visual and motor modalities: activity in visual cortex was increased with added acoustic noise, whereas activity in motor cortex was reduced.

To investigate the effect of scanner noise on verbal working memory, Tomasi et al. (2005) had participants to perform an *n*-back task using visually-displayed letters. The loudness of the EPI scanning was varied by approximately 12 dB by selecting two readout bandwidths to minimize (or maximize) the acoustic noise. No difference in behavioral accuracy was observed as a function of noise level. However, although the overall spatial patterns of task-related activity were similar, brain activity differed as a function of noise. The louder sequence was associated with increased activity in several regions including large portions of (primarily dorsal) frontal cortex and cerebellum, and the quieter sequence was associated with greater activity in (primarily ventral) regions of frontal cortex and left temporal cortex.

Behaviorally, recorded scanner noise has been shown to impact cognitive control (Hommel et al., 2012); additional effects of scanner noise have been reported in fMRI tasks of emotional processing (Skouras et al., 2013) and visual mental imagery (Mazard et al., 2002). Thus, MRI acoustic noise influences brain function across a number of cognitive domains.

It is not only the loudness of scanner noise that is an issue, but also the characteristics of the sound: whether an acoustic stimulus is pulsed or continuous, for example, can significantly impact both auditory and attentional processes. Haller et al. (2005) had participants perform a visual *n*-back task, using either a conventional EPI sequence or one with a continuous sound (i.e., not pulsed). Although behavioral performance did not differ across sequence, there were numerous differences in the detected neural response. These included greater activity in cingulate and portions of frontal cortex for the conventional EPI sequence, but greater activity in other portions of frontal cortex and left middle temporal gyrus for the continuous noise sequence. As with conventional EPI sequences, scanner noise is once again found to impact neural processing in areas beyond auditory cortex (see also Haller et al., 2009).

It is worth noting that not every study investigating this issue has observed effects of acoustic noise in non-auditory tasks: Elliott et al. (1999), using participants performing visual, motor, and auditory tasks, found that scanner noise resulted in decreased activity uniquely during the auditory condition. Nevertheless, the number of instances in which scanner noise has been found to affect neural activity on non-auditory tasks is high enough that the issue should be taken seriously: Although exactly how much of the difference in neural response can be attributed to scanner noise is debatable, converging evidence indicates that the effects of scanner noise frequently extend beyond auditory cortex (and auditory tasks). These studies suggest that (1) a lack of behavioral effect of scanner noise does not guarantee equivalent neural processing; (2) both increases and decreases in neural activity are seen in response to scanner noise; and (3) the specific regions in which noise-related effects are observed vary across study.

### Overall subject comfort and special populations

An additional concern regarding scanner noise is that it may increase participant discomfort. Indeed, acoustic noise can cause anxiety in human subjects (Quirk et al., 1989; Meléndez and McCrank, 1993), a finding which may also extend to animals. Scanner noise presents more of a challenge for some subjects than others, and it may be possible to improve the comfort of research subjects (and hopefully their performance) by reducing the amount of noise during MRI scanning. Additionally, if populations of subjects differ in a cognitive ability such as auditory attention, the presence of scanner noise may affect one group more than another. For example, age can significantly impact the degree to which subjects are bothered by environmental noise (Van Gerven et al., 2009); similarly, individual differences in noise sensitivity may contribute to (or reflect) variability in the effects of scanner noise on neural response (Pripfl et al., 2006). These concerns may be particularly relevant in clinical or developmental studies with children, participants with anxiety or other psychiatric condition, or participants who are particularly bothered by auditory stimulation.

### A cautionary note regarding interactions

One argument sometimes made in auditory fMRI studies using standard EPI sequences is that although acoustic noise may have some overall impact, because noise is present during all experimental conditions it cannot influence the results when comparing across conditions (which is often of most scientific interest). Given the ample amount of evidence for auditory-cognitive interactions, such an assumption seems tenuous at best. If anything, there is good reason to suspect interactions between acoustic noise and task difficulty, which may manifest differently depending on particular stimuli, listeners, and statistical methods (for example, univariate vs. multivariate analyses). In the absence of empirical support to the contrary, claims that acoustic noise is unimportant should be treated with skepticism.

## Solutions for auditory fMRI

**Figure 3:**
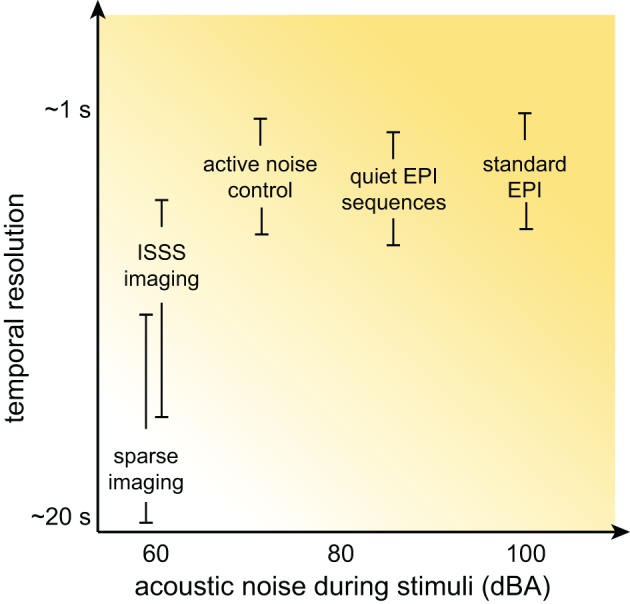
**Schematic illustration of the relationship between temporal resolution and acoustic noise during stimulus presentation for various MRI acquisition approaches**. Although the details for any specific acquisition depend on a combination of many factors, in general significant reductions in acoustic noise are associated with poorer temporal resolution.

Although at this point the prospects for auditory neuroscience inside an MRI scanner may look bleak, there is still cause for optimism. In this section I provide an overview of several methods for dealing with scanner noise that have been employed, noting advantages and disadvantages of each. These approaches are listed in Table 2, a subset of which is shown in Figure 3.

**Table 2:** **Methods for dealing with acoustic noise in fMRI**.

**Approach**	**Approximate noise reduction during stimulus (dB)^a^**	**Requires custom scanner hardware?**	**Requires custom presentation equipment?**	**Requires custom MRI sequence?**	**Image quality relative to continuous**	**Temporal resolution relative to continuous**
Continuous EPI	0	No	No	No	–	–
Passive hearing protection	35	No	No	No	No change	No change
Sparse imaging	50	No	No	No	No change	Reduced
ISSS imaging	50	No	No	Yes	No change	Slightly reduced
Active noise control	40	No	Yes	No	No change	No change
Quiet MRI sequences	20	No	No	Yes	Reduced	Slightly reduced
Scanner hardware modification	20	Yes	No	No	No change	No change

a *The actual reduction of acoustic noise can vary substantially depending on the specific equipment and implementation; these numbers are provided as a rough estimate*.

### Passive hearing protection

Subjects in MRI studies typically wear over-ear hearing protection that attenuates acoustic noise by approximately 30 dB. Subjects may also wear insert earphones, or foam earplugs that can provide additional reduction in acoustic noise of 25–28 dB, for a combined reduction of approximately 40 dB (Ravicz and Melcher, 2001). Although hearing protection can reduce the acoustic noise perceived during MRI, it cannot eliminate it completely: Even if perfect acoustic isolation could be achieved at the outer ear, sound waves still travel to the cochlea through bone conduction. Thus, hearing protection is only partial solution, and some degree of auditory stimulation during conventional fMRI is unavoidable. In addition, passive hearing protection may change the frequency spectrum of stimuli, affecting intelligibility or clarity.

### Continuous scanning using a standard EPI sequence

**Figure 4:**
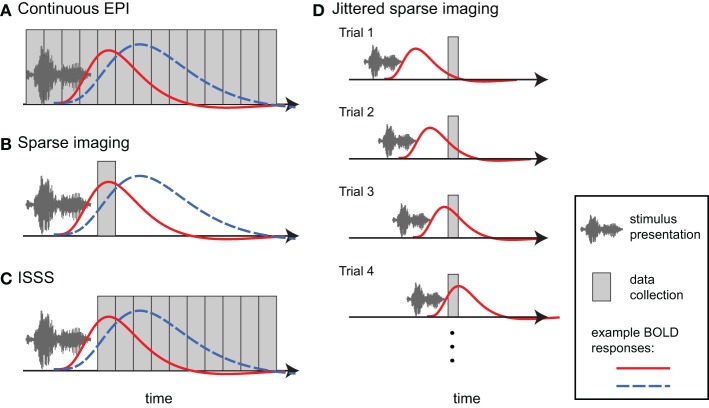
**Different approaches to imaging auditory stimuli provide varying compromises between temporal resolution and acoustic noise**. Example BOLD responses are shown in blue and red; these could reflect different responses across individuals or experimental conditions. **(A)** Continuous EPI provides relatively good temporal resolution, but with a high level of continuous acoustic noise. **(B)** Sparse imaging includes a period in which no data is collected, allowing the presentation of stimuli in relative quiet (due to the absence of gradient switching noise). The delay in the hemodynamic response enables the peak response to be collected after stimulus presentation has finished. The reduced temporal resolution of a traditional sparse imaging sequence may obscure differences in response latency or shape. In the hypothetical example, the blue response peaks higher than the red response; however, at the time when the sparse data point is collected, the red response is higher. **(C)** With interleaved silent steady state (ISSS) imaging, stimuli can also be presented in the absence of gradient switching noise, but a greater amount of data can be collected after presentation compared to sparse imaging. The delay in the hemodynamic response enables peak responses to be collected with relatively good temporal resolution. **(D)** By varying the time at which stimuli are presented relative to data collection across trials, non-continuous imaging can still provide information about the timecourse of the average response to a category of stimuli. Note how a different part of the BOLD response is sampled on each trial.

One approach in auditory fMRI is to present stimuli using a conventional continuous scanning paradigm, taking care to ensure that participants are able to adequately hear the stimuli (Figure 4A). This approach generally assumes that, because scanning noise is consistent across experimental condition, it is unlikely to systematically affect comparisons among conditions (typically what is of interest). I have already noted above the danger of this assumption with respect to additional task effects and ubiquitous interactions between perceptual and cognitive factors. However, for some paradigms a continuous scanning paradigm may be acceptable. From an imaging perspective continuous imaging will generally provide the largest quantity of data, and no special considerations are necessary when analyzing the data. Continuous EPI scanning has been used in countless studies to identify brain networks responding to environmental sounds, speech, and music. The critical question is whether the cognitive processes being imaged are actually the ones in which the experimenter is interested^2^.

### Sparse imaging

When researchers are concerned about acoustic noise in fMRI, by far the most widely used approach is sparse imaging, also referred to as clustered volume acquisition (Scheffler et al., 1998; Eden et al., 1999; Edmister et al., 1999; Hall et al., 1999; Talavage and Hall, 2012). In sparse imaging, illustrated in Figure 4B, the repetition time (TR) is set to be longer than the acquisition time (TA) of a single volume. Slice acquisition is clustered toward the end of a TR, leaving a period in which no data are collected. This intervening period is relatively quiet due to the lack of gradient switching, and permits stimuli to be presented in more favorable acoustic conditions. Because of the inherent lag of the hemodynamic response (typically 4–7 s to peak), the scan following stimulus presentation can still measure responses to stimuli, including the peak response if presentation is timed appropriately.

The primary disadvantage of sparse imaging is that due to the longer TR, less information is available about the timecourse of the response (i.e., there is a lower sampling rate). In addition to reducing the accuracy of the response estimate, the reduced sampling rate also means that differences in timing of response may be interpreted as differences in magnitude. An example of this is shown in Figure 4B, in which hemodynamic responses that differ in magnitude and timing will give different results, depending on the time at which the response is sampled.

The lack of timecourse information in sparse imaging can be ameliorated in part by systematically varying the delay between the stimulus and volume collection (Robson et al., 1998; Belin et al., 1999), illustrated in Figure 4D. In this way, the hemodynamic response can be sampled at multiple time points relative to stimulus onset over different trials. Thus, across trials, an accurate temporal profile for each category of stimulus can be estimated. Like all event-related fMRI analyses this approach assumes a consistent response for all stimuli in a given category. It also may require prohibitively long periods of scanning to sample each stimulus at multiple points; this requirement has meant that in practice varying presentation times relative to data collection is done infrequently.

Many studies incorporating sparse imaging use an event-related design, along with TRs in the neighborhood of 16 s or greater, in order to allow scanner-induced BOLD response to return to near baseline levels on each trial. Although this may be particularly helpful for experiments in which activity in primary auditory areas is of interest, it is not necessary for all studies, and in principle sparse designs can use significantly shorter TRs (e.g., <5 s). Sometimes referred to as “fast” sparse designs, sparse designs with shorter TRs enable researchers to take advantage of a faster stimulus presentation rate and acquire more data for a given period of time, and for many experiments may be a more efficient approach (Perrachione and Ghosh, 2013).

#### Cardiac gating

Cardiac gating addresses problems caused by the fact that heartbeat and associated changes in blood flow can displace brainstem structures, making activity in these regions difficult to detect. With cardiac gating, researchers monitor a subject's heart rate, and then adjust volume acquisition to be synchronized to the heart rate (i.e., occurring at a consistent time in the heart rate cycle) (Guimaraes et al., 1998). Because heart rate will not perfectly align with a chosen TR, using cardiac gating results in a variable TR (± approximately ½ heart rate). (With relatively long TRs, the variability in sampling rate is typically not a significant problem, as the response to one trial is unlikely to overlap the response to another trial). Cardiac gating reduces data variability due to cardiac pulse motion artifacts and can thus improve ability to detect activity in subcortical structures prone to these artifacts, such as the inferior colliculus and medial geniculate body (Harms and Melcher, 2002; Overath et al., 2012).

### Interleaved silent steady state (ISSS) imaging

The main disadvantages in traditional sparse imaging come from the lack of information about the timecourse of the hemodynamic response, and the relatively small amount of data collected (leading to potentially less accurate parameter estimates and fewer degrees of freedom in first-level analyses). Although in principle multiple volumes can be acquired following each silent period, the equilibrium state of the brain tissue changes during these silent periods: The additional scans do not reflect steady-state longitudinal magnetization, and thus vary over time. The lack of steady-state longitudinal magnetization adds variance to the data that can be challenging to account for in timeseries statistical models.

Schwarzbauer et al. (2006) developed a solution to this problem by implementing a sequence with continuous excitation RF pulses, but variable readout gradients. The excitation pulses maintain steady state longitudinal magnetization but produce relatively little acoustic noise. As in traditional sparse imaging, an ISSS sequence permits stimuli to be presented in quiet and the peak BOLD activity to be captured due to the delay in hemodynamic response. However, with ISSS any number of volumes can be obtained following a silent period, as illustrated in Figure 4C. Although technically the temporal resolution is reduced relative to continuous scanning—as there are times when no data is being collected—the *effective* temporal resolution can be nearly as good as continuous scanning because data collection can capture much of the BOLD response following stimulus presentation: The ability of the sequence to capture the early hemodynamic response is limited solely by the length of the stimuli (with shorter stimuli permitting data collection to start closer to stimulus onset). ISSS thus combines advantages of continuous and sparse imaging, allowing the presentation of stimuli in relative quiet, while still providing information on the timing of the hemodynamic response. Variations of ISSS fMRI have now been used successfully in numerous studies of auditory processing (Doehrmann et al., 2008, 2010; Bekinschtein et al., 2011; Davis et al., 2011; Engel and Keller, 2011; Mueller et al., 2011; Rodd et al., 2012; Yoo et al., 2012).

**Figure 5:**
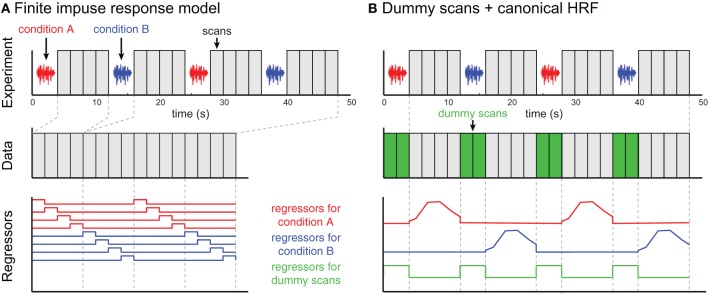
**Two examples of ISSS fMRI data analysis**. The example experiment is illustrated in the top row and identical for both approaches. No data are collected during stimulus presentation; following each silent period 4 scans are collected. **(A)** In the finite impulse response (FIR) model, scans are concatenated, and each time bin following an event is modeled using a separate regressor. The modeled scans have temporal discontinuities, but accurately represent all of the data collected. **(B)** By incorporating dummy scans in the modeled timeseries, the original temporal structure of the true data is preserved, facilitating the use of basis functions such as a canonical HRF. Regressors for experimental conditions should be set to 0 during the period of the dummy scans; the dummy scans themselves can be modeled with a single regressor. However, the modeled scans now overestimate the amount of data collected, artificially inflating the degrees of freedom in single-subject (first-level) models.

Compared to continuous or sparse imaging data, ISSS data can be challenging to analyze because the data are discontinuous—that is, the sampling rate is not consistent. Because of this added wrinkle, below I briefly review two examples of analyzing ISSS data, illustrated in Figure 5. No doubt with increasing experience ISSS data analysis can be further refined. These descriptions are based on an imaginary event-related fMRI study with two conditions (A and B) and a TR of 2 s. Each trial involves presenting a single stimulus during a period of 4 s of silence, followed by 8 s of data acquisition. With a TR of 2 s, this results in 4 volumes of data per trial.

#### Analyzing ISSS fMRI data using a finite impulse response (FIR) model

Perhaps the most straightforward approach to analyzing ISSS fMRI data is to use a finite impulse response (FIR) model, shown in Figure 5A. A typical FIR model would consider only the scans on which data was collected. The model would thus have 4 regressors for condition A (one for each volume following stimulus presentation), and 4 regressors for condition B. These regressors would model the response at each time bin following a stimulus, making no assumptions about the shape of the response. As with any FIR Model, given the multiple regressors for each condition, there are several ways of summarizing the response to a condition, including an *F*-test over all 4 columns for a condition (asking: is there any effect at any time point?) or a *t*-test over all 4 columns (on average is there an increased response?). Similar options exist for comparing response between conditions.

Because the ISSS scans are not continuous, care must be taken when implementing temporal filtering, including typical highpass filtering done on fMRI timeseries data. Omitting highpass filtering may make an analysis particularly susceptible to the influence of low-frequency (non-acoustic) noise. One way to help mitigate this issue is to ensure trials of different conditions are not too far apart in time so that comparisons across conditions are not confounded with low-frequency fluctuations in the signal.

#### Analyzing ISSS fMRI data using dummy scans to mimic a continuous timeseries

An alternative approach is to ensure that rows of the design matrix correspond to a continuous timeseries, illustrated in Figure 5B. To accomplish this, dummy volumes can be included in the design matrix during the period in which no data were actually collected. A straightforward option is to use the mean EPI image across all (real) volumes in a session, although any identical image will work: Using an identical image for all dummy images means that all dummy images can be perfectly modeled using a single regressor (0 for real scans, 1 for dummy scans). With this model it is then possible to use a canonical HRF (or any other basis set) for events of interest; the parameter estimates for these regressors are not influenced by the dummy scans. It is important to set the values for the non-dummy regressors to zero during the dummy scans to preserve estimation of the parameter estimate, and to rescale the regressors so that the maximum values are matched after these adjustments.

It is not actually necessary to use dummy scans in order to take advantage of timeseries properties, such as highpass filtering or using an informed basis function (e.g., a canonical HRF); an appropriate design matrix that takes into account the discontinuous nature of the data could be constructed. However, the use of dummy scans facilitates constructing design matrices within common fMRI analysis software packages, which are typically designed to work with continuous timeseries data.

When dummy scans are included in the final design matrix, the default degrees of freedom in the model will be incorrectly high, as the dummy scans should not be counted as observations. Thus, for first-level (single subject) analyses, an adjustment to the degrees of freedom should be made for valid statistical inference. For group analyses using a two-stage summary statistics procedure, however, adjusting for first-level degrees of freedom is not necessary.

### Active noise control

A different approach to reducing the impact of acoustic noise in the MRI scanner is to change the way this sound is perceived by listeners using active noise control (Hall et al., 2009). As typically implemented, active noise control involves measuring the properties of the scanner noise, and generating a destructive acoustic signal (also known as “antinoise”) which is sent to the headphones that cancels a portion of the scanner noise (Chambers et al., 2001, 2007; Li et al., 2011). The destructive signal is based on estimates of scanner noise that can either be fixed, or adjusted over the course of a scanning session to accommodate changes in the scanner noise. Adjusting over time may be important in the context of fMRI as subjects may move their heads over the course of a scanning session, which affects the acoustic characteristics of the noise reaching their ears.

In addition to sound presentation hardware, active noise control also requires an MR-compatible method for measuring the acoustic noise in the scanner, used to shape the destructive noise pulses. Whether sound is generated in the headset, or passed through a tube, the timing of this canceling sound is critical, as it must arrive with the specified phase relationship to the scanner noise.

Active noise control can reduces the level of acoustic noise by 30–40 dB, and subjective loudness by 20 dB (the difference between these measures likely reflecting the contribution of bone conducted vibration) (Hall et al., 2009; Li et al., 2011). Particularly relevant is that when using relatively simple auditory stimuli (pure tone pitch discrimination), (1) behavioral performance in the scanner was significantly better and (2) activity in primary auditory regions was significantly greater under conditions of active noise control compared to normal presentation (Hall et al., 2009).

### Using continuous fMRI sequences with reduced acoustic noise

Software modifications to EPI sequences intended to reduce the effects of acoustic scanner noise can be broadly grouped into two approaches: changing the nature of the acoustic stimulation and reducing the overall sound levels.

One approach to reducing sound levels of a standard EPI sequence is to modify the gradient pulse shape (Hennel et al., 1999). Typically, gradient pulses are trapezoidal, to increase the speed and efficiency of gradient encoding. By using sinusoidal pulses, acoustic noise can be reduced during BOLD fMRI (Loenneker et al., 2001), with some increase in the spatial smoothness of the reconstructed data.

Building on the idea of modified pulse shape, another type of quiet fMRI sequence was introduced by Schmitter et al. (2008). Their quiet continuous EPI sequence takes advantage of two key modifications to reduce acoustic noise. The first involves collecting data using a sinusoidal traversal of *k* space, enabling more gradual gradient switching (readout gradients are purely sinusoidal) and reducing the acoustic noise produced. The second modification addresses the fact that a large component of the acoustic noise during EPI comes from the resonance of the scanner hardware to the gradient switching. This reflects specific physical properties of each scanner, and varies across different speeds of gradient switching. Thus, it is possible to perform scanner-specific measurements of the acoustic noise generated for different readout gradient switching frequencies, and select a combination of parameters that is relatively quiet, but does not unacceptably compromise signal quality. In Peelle et al. (2010a), we chose a bandwidth of 1220 Hz/Px and an echo time of 44 ms (compared to a standard sequence with values of 2230 Hz/Px and 30 ms, respectively). As might be expected, the longer echo time lead to moderate increases in signal dropout in regions prone to susceptibility artifact, such as inferior temporal and ventromedial frontal cortex. Together, these modifications produce approximately a 20 dB reduction in acoustic noise for the scanner, and using this sequence results in greater activity in several auditory regions compared to a standard continuous sequence (Schmitter et al., 2008; Peelle et al., 2010a).

Taking another approach to reducing the impact of scanner noise on observed activation, Seifritz et al. (2006) developed a continuous-sound EPI sequence to reduce the auditory stimulation caused by rapid acoustic pulses (Harms and Melcher, 2002), as found in conventional EPI. In their sequence the RF excitation pulses, phase-encoding gradients, and readout gradients are divided into short trains. The resulting repetition rate is fast enough that the acoustic noise is perceived as a continuous sound, rather than the pulsed sound perceived in conventional EPI. Using sparse imaging, the authors compared neural activity in response to audio recordings of conventional EPI compared to the “continuous sound” sequence. They found that the continuous sound sequence resulted in reduced activity in auditory cortex due to scanner noise, and increased activity to experimental manipulations.

### Scanner hardware modification

Although it may not be practical for most research groups to significantly modify scanner hardware, by changing the physical configuration of the MRI scanner it is possible to significantly reduce the amount of acoustic noise generated. Some approaches have included the use of rotating coils to reduce gradient switching (Cho et al., 1998b), placing the gradient coils in a vacuum to reduce noise propagation (Katsunuma et al., 2001), or altering the coil structure (Mansfield and Haywood, 2000). By combining multiple approaches and focusing on the largest contributors to acoustic noise, substantial reductions in noise levels can be achieved (Edelstein et al., 2002). In the future, commercial applications of these approaches may help to limit the impact of scanner noise during fMRI, particularly when combined with some of the other solutions outlined above.

### Auditory fMRI in nonhuman animals

Although my focus has been on fMRI of human subjects, many of these same challenges and solutions apply equally when using fMRI with animals (Petkov et al., 2009; Hall et al., 2014). As with human listeners, the choice of scanning protocol will depend on a researcher's primary interests and the acceptable level of tradeoff between data quality, temporal resolution, and acoustic noise. Although some concerns about attention and cognitive challenge may be mitigated when dealing with sedated animals, in the absence of empirical support it is probably not safe to assume that one protocol will prove optimal in all situations. In addition, the timing parameters of any non-continuous sequence will naturally need to be optimized for the HRF of the animal being studied (Brown et al., 2013).

### Choosing the appropriate solution

**Figure 6:**
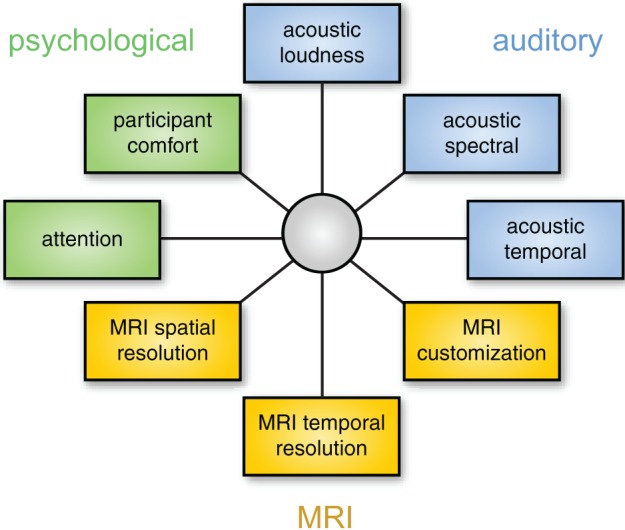
**Choosing the best method for auditory fMRI involves considering a number of dimensions**. These dimensions are not independent: for example, using a modified EPI sequence may change the properties of the MRI data, the acoustic properties of the scanning noise, and resulting impact on psychological processes. The focus of optimization will depend on the acoustic characteristics of the stimuli and the neural processes of interest.

As discussed above, different solutions for auditory fMRI have intrinsic strengths and weaknesses, and thus any chosen approach involves a degree of compromise with respect to acoustic noise (loudness or quality), psychological impact, and MRI data characteristics. It may be useful to think about this in a framework of multidimensional optimization, as illustrated in Figure 6. Because these dimensions are not independent, it is impossible to optimize for everything simultaneously (for example, approaches that have the lowest acoustic noise also tend to have poorer temporal resolution, forcing a researcher to choose between noise level and temporal resolution). It is therefore important to identify the dimensions that are most important for a given study. These will depend on the specific stimuli and scientific question at hand.

Although there are exceptions, as a general rule it is probably safest to prioritize the auditory and psychological aspects of data collection. If the processing of stimuli is affected by scanner noise (through masking or increased perceptual effort), the resulting neural processing may differ from what the researchers are interested in. In this case increased image quality will not help in identifying neural activity of interest. Thus, a sparse imaging sequence is nearly always preferable to continuous sequences because it presents the lowest level of background noise, and is straightforward to implement. If possible, an ISSS sequence presents an even stronger solution as it permits the presentation of stimuli in relative quiet, while not sacrificing temporal resolution to the same degree as a traditional sparse sequence.

When it is not feasible to present stimuli in the absence of scanner noise, considering the acoustic characteristics of the stimuli is critical. For example, if speech prosody, voice/speaker perception, or musical timber is of interest, spectral cues may be particularly important, and thus the spectrum of the scanner noise may be a deciding factor. In contrast, for other stimuli (such as musical beat, or other aspects of speech perception) temporal factors may dominate.

That being said, from a practical standpoint the majority of researchers will be constrained by available sequences and equipment, and thus the most common choice will be between a continuous EPI sequence and a traditional sparse sequence. In this case, adapting a paradigm and stimuli to work with the sparse sequence is almost always a safer choice.

### Relying on converging evidence to support conclusions

Although it is no doubt important to optimize fMRI acquisition and analysis parameters for auditory studies, the strongest inferences will always be drawn based on converging evidence from multiple modalities. With respect to auditory processing, this includes functional neuroimaging methods that allow the measuring of neural response in the absence of external noise such as positron emission tomography (PET), electroencephalography (EEG), magnetoencelphalography (MEG), electrocorticography (ECoG), or optical imaging, as well as studying behavior in people with differences in brain structure (e.g., as a result of stroke or neurodegenerative disease).

## Conclusions and recommendations

Echoplanar fMRI is acoustically noisy and poses considerable challenges for researchers interested in studying auditory processing. Although it is impossible to fully resolve the tension between the acoustic noise produced during fMRI and the desired experimental environment, the following steps will often be helpful in optimizing auditory responses and our interpretation of them:

1. Address, rather than ignore, the possible effects of background noise on activity seen in fMRI studies. Considering scanner noise is particularly important when using auditory stimuli, but may apply to non-auditory stimuli as well.
2. When possible, use methods that limit the impact of acoustic noise during fMRI scanning.
3. Provide empirical demonstrations of the effect of scanner noise on specific paradigms and analyses.

It is an exciting time for auditory neuroscience, and continuing technical and methodological advances suggest an even brighter (though hopefully quieter) future.

### Conflict of interest statement

The author declares that the research was conducted in the absence of any commercial or financial relationships that could be construed as a potential conflict of interest.

## Footnotes

^1^Noise vocoding (Shannon et al., 1995) involves dividing the frequency spectrum of a stimulus into bands, or channels. Within each channel, the amplitude envelope is extracted and used to modulate broadband noise. Thus, the number of channels determines the spectral detail present in a speech signal, with more channels resulting in a more detailed (and for speech, more intelligible) signal (see Figure 2 in Peelle and Davis, 2012).

^2^For researchers who question whether acoustic noise during fMRI may impact cognitive processing, it may be interesting to suggest to a cognitive psychologist that they play 100 dB SPL sounds during their next behavioral study and gauge their enthusiasm.
